# Management of carotid cavernous fistulas: A single center experience

**DOI:** 10.3389/fneur.2023.1123139

**Published:** 2023-02-09

**Authors:** Georgios Luca Alatzides, Marcel Opitz, Yan Li, Sophia Goericke, Marvin Darkwah Oppong, Benedikt Frank, Anja Katrin Eckstein, Martin Köhrmann, Karsten Wrede, Michael Forsting, Isabel Wanke, Cornelius Deuschl

**Affiliations:** ^1^Institute of Diagnostic and Interventional Radiology and Neuroradiology, Essen University Hospital, Essen, Germany; ^2^Department of Neurosurgery and Spine Surgery, Essen University Hospital, Essen, Germany; ^3^Department of Neurology and Center for Translational Neuro- and Behavioral Sciences (C-TNBS), Essen University Hospital, Essen, Germany; ^4^Department of Ophthalmology, Essen University Hospital, Essen, Germany; ^5^Department of Neuroradiology, Klinik Hirslanden and Swiss Neuro Radiology Institute, Zurich, Switzerland

**Keywords:** carotid cavernous fistula (CCF), dural arteriovenous fistula (DAVF), diagnostic cranial angiography (DCA), endovascular treatment (EVT), coils, liquid embolics

## Abstract

**Purpose:**

Multimodal endovascular therapy (EVT) of carotid cavernous fistula (CCF) with different approaches and a variety of available embolization material enable high occlusion rates with good clinical and functional outcome but until now there is still little evidence available. This retrospective single-center study aims to evaluate EVT of CCF with different neuroendovascular techniques regarding occlusion rates, complications and outcomes.

**Materials and methods:**

From 2001 to 2021 59 patients with CCF were treated at our tertiary university hospital. Patient records and all imaging data including angiograms were reviewed for demographic and epidemiological data, symptoms, fistula type, number of EVTs, complications of EVT, type of embolic materials, occlusion rates and recurrences.

**Results:**

Etiology of the CCF were spontaneous (41/59, 69.5%) post-traumatic (13/59, 22%) and ruptured cavernous aneurysms (5/59, 8.5%). Endovascular therapy was completed in one session in 74.6% (44/59). Transvenous access was most frequent (55.9% 33/59) followed by transarterial catheterization in 33.9% (20/59) and a combination of both (6/59, 10.2%). Exclusively coils were used in 45.8% (27/59), a combination of ethylene vinyl alcohol (EVOH) copolymer (Onyx) and coils in 42.4% (25/59). Complete obliteration was achieved in 96.6% of patients (57/59) with an intraprocedural-related complication rate of 5.1% (3/59) and no mortality.

**Conclusion:**

Endovascular therapy of CCF has been shown to be safe and effective with high cure rates and low rates of intraprocedural complications and morbidity even in complex scenarios.

## Introduction

Carotid cavernous fistulas (CCF) are abnormal vascular shunts between the carotid arterial system with its branches and the cavernous sinus venous system. They can be either divided based on their angiographic architecture into direct and indirect CCF according to the Barrow classification system or based on their etiology into spontaneous, traumatic or in conjunction with an aneurysm in the cavernous segment of the ICA (1–3). Transmission of highly pressurized arterial blood into the cavernous sinus leads to venous hypertension with retrograde ophthalmic and sometimes cortical venous flow which is reflected by typical signs and symptoms of CCF such as proptosis, conjunctival injection, visual loss, cranial nerve deficits, pulsatile tinnitus and more rarely intracranial hemorrhage and increased intracranial pressure (2, 4–6).

Conservative management with manual external compression of the cervical carotid artery several times a day might be an effective treatment option when the fistula exhibits low flow characteristics and there is no cortical venous drainage or progressive visual or neurological decline (7). In nearly all other cases fistula occlusion is the treatment objective, while maintaining normal flow through the internal carotid artery (ICA) (2, 8, 9).

In the 1970s endovascular procedures mainly consisted of transarterial application of a detachable balloon to occlude the fistula but endovascular techniques and their efficacy and safety rapidly evolved since then. Modern endovascular therapy offers multimodal treatment strategies through transarterial or transvenous approaches with a variety of embolization materials with detachable coils and liquid agents such as n-butylcyanoacrylate (n-BCA) or ethylene vinyl alcohol (EVOH) polymer (Onyx^®^, Medtronic, Inc., Irvine, USA) becoming the primary tools (3, 10, 11). Under certain circumstances, e.g. severe arterial injury with large wall defects, the additional use of balloon protection of the ICA or a stent is indicated for endoluminal reconstruction or to prevent protrusion from embolic material into the lumen of the parent vessel to avoid the risk of causing stroke or fistula recurrence (10, 12, 13). When there is extensive vessel damage and no prospect of achieving endovascular fistula occlusion with preservation of the ICA, arterial sacrifice by complete ICA occlusion or surgical management *via* different methods including suturing, clipping or packing the fistula and/or cavernous sinus remain last resort treatment options (4, 10, 14, 15).

Nevertheless, there is still inhomogeneous consensus and vivid debate regarding the rational use of these developments, so that we aimed to investigate periprocedural aspects of the multimodal endovascular techniques, their safety and efficacy in patients with CCF treated at our institution during the last 20 years.

## Materials and methods

### Study design

We conducted a retrospective observational study at a tertiary neuroendovascular care center. The study was approved by the local Ethics Committee and conducted in accordance with the principles of the Declaration of Helsinki. By reviewing our database, all patients with CCF who underwent an endovascular procedure between 2001 and 2021 were identified and screened for eligibility.

Clinical data, such as demographics and symptomatology, fistula morphology, periinterventional imaging and procedure reports, number of endovascular sessions as well as angiographic and short-term clinical outcome—if available—were collected. Procedure-associated complications were categorized in overall-related and direct intraprocedural-related complications.

In a few cases, specific symptomatology was not evaluable due to a comatose state of patients upon hospital admission. We exclusively focused on CCF-related clinical symptoms. Eight patients were excluded due to missing periprocedural data and one patient because of prior endovascular therapy of a CCF (Supplementary material 1). All CCF were classified according to the Barrow classification (1). Barrow type A fistulas were depicted as direct fistulas whereas type B–D constituted the group of indirect fistulas. A defined follow-up regime did not exist, follow up data were gathered within one year after the intervention.

### Endovascular procedure

**Figure 1:**
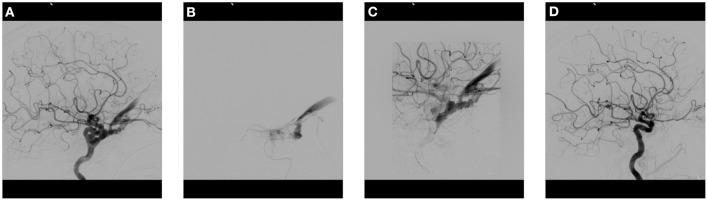
Cranial angiogram of a patient with sudden onset of bulbar protrusion, chemosis and visual loss after a fall with skull base fracture. **(A)** Lateral DCA image of the left ICA demonstrates a direct CCF with massive arterialization of the cavernous sinus and dilatation of the superior ophthalmic vein and inferior petrosal sinus. **(B)** Transarterial placement of a microcatheter in the cavernous sinus. **(C)** Progressive filling of the cavernous sinus with multiple coils. **(D)** Post-therapeutic lateral control angiogram of the ICA demonstrates complete fistula occlusion without retrograde venous filling. CCF, Carotid cavernous fistula; DCA, Diagnostic cranial angiography; ICA, Internal carotid artery. (see legend in Figure 2).

**Figure 2:**
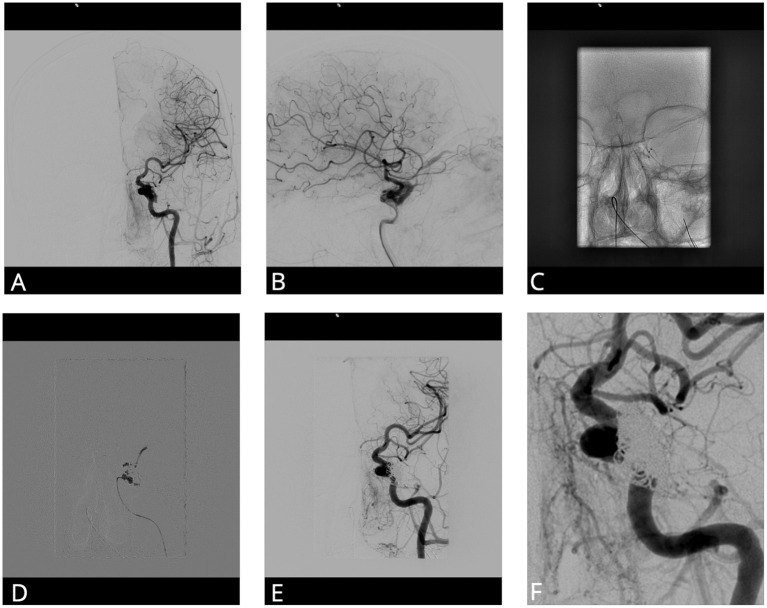
Cranial angiogram of a patient with progressive headache and visual deficits. **(A, B)** Anteroposterior and lateral DCA image after injection of the left ICA shows a CCF with origin of the lateral wall of the cavernous part of the ICA. **(C)** Two microcatheters in the cavernous sinus *via* a transvenous route. **(D)** Application of 0.44 ml of Onyx after filling the cavernous sinus with some coils. **(E, F)** Control anteroposterior angiogram shows complete fistula occlusion. CCF, Carotid cavernous fistula; DCA, Diagnostic cranial angiography; ICA, Internal carotid artery.

In most of the cases patients received diagnostic cranial angiography (DCA) prior to embolization, whereby DCA was performed immediately before the neurointerventional procedure or as a stand-alone diagnostic procedure. This was performed to confidently make the diagnosis of a CCF, characterize the vascular architecture along with the venous drainage pattern according to the Barrow classification and detect important accompanying factors such as cortical venous drainage or presence of an intracranial aneurysm. Every case was individually evaluated in an interdisciplinary exchange between neurosurgeons and interventional neuroradiologists. The definitive endovascular treatment strategy with its approach (transarterial vs. transvenous vs. combined) and used embolization material depended on the fistula morphology and the preference of the neurointerventionalist (exemplary cases Figures 1, 2). In the majority of the cases (42/59, 71.2%) post-procedural cross sectional imaging *via* CT or MRI to rule out any periprocedural complication was available in our database.

### Statistical analysis

Analyses were conducted to detect potential correlations regarding the clinical data, fistula morphology, periprocedural data, complications, and outcome of the patients of the study cohort.

Continuous data were evaluated for normality of distribution using the Kolmogorov Smirnov test and by inspection of the plots. Continuous variables are summarized as mean (standard deviation) in case of normal distribution, otherwise as median (interquartile range) and categorical variables as count (percentage). The Chi- square test for trend and Fisher's exact test were used for testing association between categorical and the Mann-Whitney-*U*-test or *t*-test for continuous variables. The level of significance was set to 0.05. All analyses were performed using SPSS (IBM Corp., SPSS Statistics, Version 27.0. Armonk, NY).

## Results

### Clinical data and fistula morphology

Overall, 59 patients were enrolled for this retrospective study after excluding 8 patients not fulfilling the inclusion criteria (Supplementary material 1). Among them, 17 were males (28.8%) and 42 females (71.2%). Median age of the study cohort was 71.0 years (IQR 20.0). On hospital admission visual symptoms such as orbital chemosis, diplopia, proptosis or visual loss were observed most commonly (48/50, 96.0%), whereas less than half of the included patients presented with headache (20/50, 40.0%), tinnitus (9/50, 18.0%) and only two patients with focal neurological deficits (2/50, 4.0%). A spontaneous etiology of CCF was seen most often (41/59, 69.5%) followed by traumatic CCFs (13/59, 22.0%) with predominantly an acute trauma history (8/13, 61.5% vs. 5/13, 38.5). In five patients an intracranial aneurysm of the ICA led to formation of a CCF (5/59, 8.5%) with rupture in three cases (3/5, 60.0%). The most common type of CCF in our cohort were indirect type D fistulas (29/59, 49.2%), followed by direct type A fistulas (25/59, 42.4%). Isolated indirect fistulas of the ICA, namely type B and the corresponding counterpart of the ECA, namely type C were observed less often (2/59, 3.4% and 3/59, 5.1%, respectively). In nearly one third of patients our study cohort, a cortical venous drainage pattern could be detected (18/59, 30.5%) (Table 1).

**Table 1:** Clinical data and fistula morphology.

	**Entire cohort *n* = 59**
**Clinical data**	
Age, (years)^*^	71.0 (20.0)
Male gender	17 (28.8)
Clinical presentation	50 (84.7)
Headache	20 (40.0)
Ocular, visual	48 (96.0)
Pulse synchronous tinnitus	9 (18.0)
Focal neurological deficits	2 (4.0)
**Etiology**	
Spontaneous	41 (69.5)
Trauma	13 (22.0)
Acute ( ≤ 10 days)	8 (61.5)
Subacute (>10 days)	5 (38.5)
Aneurysm	5 (8.5)
Ruptured	3 (60.0)
Non-ruptured	2 (40.0)
**Fistula morpholgy**	
Barrow classification	
A	25 (42.4)
B	2 (3.3)
C	3 (5.1)
D	29 (49.2)
Cortical venous drainage	18 (30.5)

Values represent as count (percentage).

^*^Values represent median (IQR).

### Periprocedural aspects and used material

In 74.6% (44/59) of the embolization procedures one session was sufficient while in 25.4% (15/59) more than one session was necessary for adequate fistula occlusion. No patient required more than two sessions for adequate fistula occlusion or reduction. Most of the procedures were performed *via* a transvenous approach (33/59, 55.9%), followed by a transarterial access (20/59, 33.9%). A combined transarterial/-venous approach was used in 6/59 patients (10.2%). For one patient (1/59, 1.7%) a transorbital approach *via* the angular and superior ophthalmic vein (SOV) was used for access to the CCF as the fistula could not be treated on the conventional accesses.

CCF occlusion was achieved with solely coils (27/59, 45.8%) or coils in combination with liquid embolic agents (25/59, 42.4%). In five patients (5/59, 8.5%) we used liquid embolization agents solely and in two cases (2/59, 3.4%) a permanent stent graft (Leo stent, Balt, Montmorency, France and Jomed Mastergraft, Jomed International, Helsingborg, Sweden) was implanted. In 15 cases (15/59, 25.4%) a temporary assist device for protection of the ICA was brought in Table 2.

**Table 2:** Periprocedural data & embolization material.

	**Entire cohort *n* = 59**
**Procedural data**	
Number of sessions	
One-stage	44 (74.6)
Two-stage	15 (25.4)
Approach	
Transarterial	20 (33.9)
Transvenous	33 (55.9)
Combined	6 (10.2)
Additional transorbital	1 (1.7)
**Material**	
Onyx alone	4 (6.8)
PVA particles	1 (1.7)
Coils alone	27 (45.8)
ICA sacrifice	3 (5.1)
Onyx + Coils	25 (42.4)
Stent	2 (3.4)
Assist devices	
No	44 (74.7)
Stent assisted	5 (8.5)
Balloon assisted	10 (16.9)

Values represent as count (percentage).

PVA, Polyvinyl alcohol particles.

Differentiating the embolization material in relation to the etiology of the CCF there was no significant difference between these groups (*p* = 0.45): A combination of coils and liquid embolic material was used in most cases when the CCF was of spontaneous origin (19/41, 46.3%). Most of traumatic CCFs were treated *via* coils alone (8/13, 61.5%). In two patients with CCF based on an ICA aneurysm, obliteration of the aneurysm itself and the fistula could be achieved *via* coils leading to occlusion of the fistula (2/5, 40%), whereas in the remaining cases, flow diverter and/or additional liquid embolic (Onyx)—injected under balloon protection—were applied (3/5, 60.0%). In one of those cases, adequate treatment was not possible with preservation of the ICA so that the ICA was completely coiled and occluded.

Temporary assist devices, mainly balloon protection systems were used significantly more often in CCFs with a traumatic etiology (8/13, 61.5%) (*p* < 0.01). Looking at the embolization material used based on the fistula angioarchitecture, most of Barrow type A fistulas were obliterated *via* the sole use of coils (17/25, 68.0%), while type D fistulas were mostly treated with a combination of coils and liquid embolics (17/29, 58.6%) followed by the sole use of coils (8/29, 27.6) (*p* < 0.01). Eleven patients with a fistula type A received an assist device (11/25, 44.0%), while this rate was lower in patients with type D fistula (3/29, 10.3%) (*p* = 0.03) (Table 3).

**Table 3:** Embolization material used regarding CCF etiology and fistula morphology.

		**Material**				
	**Liquid embolics alone**	**Coils alone**	**Combined**	***p*** **-value**	**Assist devices (stent/ballon assisted)**	***p*** **-value**
**Etiology**				0.45		< 0.01
Spontaneous (*n* = 41)	5 (12.2)	17 (41.5)	19 (46.3)		3 (7.3)	
Traumatic (*n* = 13)	0 (0.0)	8 (61.5)	5 (38.5)		8 (61.5)	
Aneurysmatic (*n* = 5)	0 (0.0)	2 (40.0)	3 (60.0)		4 (80.0)	
**Barrow classification**				< 0.01		0.03
A (*n* = 25)	0 (0.0)	17 (68.0)	8 (32.0)		11 (44.0)	
B (*n* = 2)	1 (50.0)	1 (50.0)	0 (0.0)		1 (50.0)	
C (*n* = 3)	1 (33.3)	1 (33.3)	1 (33.3)		0 (0.0)	
D (*n* = 29)	3 (10.3)	8 (27.6)	17 (58.6)		3 (10.3)	

Values represent as count (percentage).

### Angiographic/clinical outcomes

Most of the patients experienced a significant improvement of their CCF-related symptoms (40/44, 90.9%), whereas four patients (4/44, 9.1%) showed a stable or undulating course with overall minor improvements. Three patients remained with a residual abducens nerve palsy although other CCF-related symptoms such as headache, diplopia and an oculomotor nerve palsy disappeared in the follow up examination. One of those patients experienced a rupture of a cavernous ICA aneurysm that showed minimal but residual inflow after the endovascular coiling procedure while a complete fistula occlusion was achieved. In another case, a traumatic event led to CCF formation with initially subclinical development of symptoms and significant progression of diplopia in the subacute setting.

Another patient showed persistent elevated intraocular pressure and a ciliary injection, although a complete endovascular fistula occlusion was achieved. None of the patients in our study cohort experienced a worsening of their symptoms post-interventional (Table 4).

**Table 4:** Outcome and complications.

	**Entire cohort *n* = 59**
**Outcome**	
Angiographic	
Complete obliteration	57 (96.6)
Partial obliteration	2 (3.4)
(Residual inflow not completely ruled out)	
Failed obliteration/Recanalization	0 (0)
Post-interventional cross sectional imaging	42 (71.2)
Complete obliteration	35 (83.3)
Partial obliteration	7 (16.7)
Failed obliteration/Recanalization	0 (0)
Clinical	44 (74.6)
Significantly improved	40 (90.9)
Stable, undulating	4 (9.1)
Worsened	0 (0)
**Complications**	
Overall procedure-related complications	6 (10.2)
Severity	
Clinically not significant	1 (16.7)
Clinically significant	5 (83.3)
Kind of complication	
Extravasation of embolic agent	1 (16.7)
Thrombosis, Infarct, spasm	2 (33.3)
Local, other	3 (50.0)

Values represent as count (percentage).

Angiographic examination in the same session after the therapeutical procedure or in the further short-term course revealed a successful complete fistula occlusion in most patients (57/59, 96.6%). In two cases (2/59, 3.4%) a partial obliteration with minor residual flow through the fistula was seen in the immediate post-interventional angiography. A detailed description of these two patients is depicted in the Supplementary material 2.

When a post-interventional cross sectional imaging, such as CT or MRI, either in the same hospital stay or during short-term follow up was conducted (*n* = 42), complete fistula occlusion was detected in 83.3% (35/42) while in the remaining cases a partial obliteration with minimal residual fistula inflow could not be ruled out completely (7/59, 16.7%) (Table 4). Among patients with an aneurysmatic origin of the CCF, one patient (1/5, 20.0%) showed residual inflow of the fistula.

### Complications

A detailed description of the observed periprocedural complications is depicted in Supplementary material 3.

Overall, we registered a complication rate of 10.2% (6/59), whereas in the majority of cases these complications were clinically significant in the short term follow up of the patients (5/6, 83.3%). An arterial vascular problem such as thrombosis, spasm and consecutive infarction led to an unfavorable event in two of those cases (2/6, 33.3%). In one combined approach without intraprocedural heparin administration slight dislocation of the inserted microcatheter led to a tiny dissection of the ICA and intraprocedural M1-occlusion that was fully recanalized after a single aspiration maneuver. Complete fistula occlusion with the combined use of coils and liquid embolics under balloon protection was not achieved in this case. The other vascular-related intraprocedural complication was observed in the treatment of bilateral traumatic CCFs *via* a transarterial approach with intraprocedural administration of heparin (5000 IU). An embolic occluded frontal branch of the ipsilateral middle cerebral artery was not fully recanalized with the use of 9 mg of Abciximab and 40 mg Alteplase. Post-interventional cross sectional imaging revealed infarction of the corresponding area. In one case extravasation of the used liquid embolic agent resulted in thrombosis of the superior ophthalmic vein with consecutive ocular symptomatology (Table 4, Supplementary material 3). In regard of the complication rate in subgroups of the study cohort there was no significant differences between direct and indirect CCF (*p* = 0.64) as well as different vascular routes (*p* = 0.13, Supplementary material 4).

## Discussion

In this retrospective, observational single center study at a tertiary neurovascular center we aimed to investigate peri- and intraprocedural aspects of the endovascular treatment of patients with CCF. We could demonstrate that high fistula occlusion rates are feasible with only low periprocedural complication rates in partially complex cases. Using a variety of strategies with different embolic materials, assist devices and techniques even fistulas with complex angioarchitecture could be treated successfully. Different vascular approaches seemed to be promising in certain anatomic and clinical situations whereby the more invasive, direct transorbital access was only seldom in our study cohort. Nevertheless, it must be stated that—although rare—peri- and intraprocedural complications had significant implications on the affected patients.

Technical applicability of transarterial embolization with detachable platinum coils evolved rapidly over time and has been shown to provide satisfactory results regarding angiographic and clinical outcomes so that endovascular coiling of CCF became the procedure of choice after largely withdrawing detachable balloons from the market (2, 4, 13). The advantage of coils is their versatile use in different approaches and the fact that they can be repositioned if their initial placement is not optimal (16). Nonetheless coil dislocation or incomplete fistula embolization because of compartmentalization within the cavernous sinuous when dense packing with a high number of coils is needed, are potential disadvantages (3, 16). We used coils as the only embolization material in 27 cases of our study cohort whereby most patients with a traumatic origin and a Barrow type A fistula were treated *via* this way. Due to their injury pattern direct type A fistulas are sometimes accompanied with dissections and lacerations of the ICA whereby a sudden increase of the intraluminal vessel pressure might play an important role (2, 12, 17). Under these circumstances assist devices such as balloons and stents are required to enable endoluminal reconstruction and protect the parent vessel. In addition, they facilitate the deposition of multiple coils in the cavernous sinus therefore reduce the risk of a potential coil protrusion into the ICA, especially in cases where a high number of coil material or rather a pronounced coil length is necessary (3, 10, 18).

Implantation of a permanent covered stent with consecutive vessel sacrifice of the ICA happened in one case with a giant aneurysm of the ICA and another case where fistula occlusion *via* a transarterial and transvenous route was not achievable. Both patients showed rapid improvement of their clinical symptoms and angiographically complete occlusion of their CCF. In the other few cases with emergency sacrifice of the parent artery, complete ICA occlusion was done *via* the sole use of coils (3/59, 5.1%). Due to the multimodal treatment options with their different approaches and techniques we could therefore reduce the rate of parent vessel sacrifice to adequately reach a satisfactory fistula reduction when comparing with other studies (19, 20).

Especially in indirect CCF with complex angioarchitecture, difficult vessel access and feeding vessels of smaller caliber liquid embolics such as n-BCA or Onyx seem to have their advantages while allowing a better penetration into these vessels in comparison with coils or other embolization materials (21–23). Gandhi et al. also demonstrated, that—although not routinely used as the first choice—transarterial embolization of indirect CCF with Onyx in certain complex scenarios might be a valuable option (24). In addition, Onyx has its legitimacy in traumatic CCF and as an additional component with other embolization materials (4, 25). Due to its non-adhesive characteristics, Onyx is more cohesive and can therefore be injected with a higher volume and at a slower rate as well as discontinuously, thus resulting in improved accuracy (16). The risk of a potential catheter retention due to fixing the catheter is reduced in comparison with other liquid embolics such as n-BCA (23). The most used liquid embolic material in our study cohort was Onyx whereby only in a minority of patients Onyx was used solely (4/59, 6.8%).

Zaidat et al. could demonstrate that a combination of Onyx and coils was able to reach high fistula occlusion rates. They postulated that penetration of the liquid agent into the matrix of the coil mass enables complete obliteration with less glue material (3). Alexander and colleagues found out that in their study cohort of patients with indirect CCF the complication rate was significantly lower when there was a combined use of Onyx and coils instead of Onyx as the sole embolic agent (26). We described a combination of those two materials in 25 cases of our study cohort (25/59, 42.4%). Regardless of the fistula origin or vascular subtype this treatment modality could achieve a complete fistula rate in most of the cases (24/25, 96.0%).

Features of dural CCFs with multiple tiny or tortuous vessels impede a rapid and successful fistula obliteration *via* a transarterial approach so that the transvenous route *via* different pathways is preferred in most of those cases (16). Although actual data claimed that—in patients with CCF and cavernous sinus dural arteriovenous fistula—the transvenous route *via* the inferior petrosal sinus (IPS) seems to be the most effective method in regard of fistula occlusion and periprocedural complication rates, a current systematic literature review could not confirm this: after evaluating 57 studies including 1,575 patients with CCF Texakalidis et al. rather stated no significant difference in the effectiveness of transarterial and transvenous embolization with an overall low complication risk of all endovascular procedures in direct and indirect CCF (26–28).

In accordance with current concepts of endovascular embolization of CCF we also used a transvenous approach more frequently than a transarterial approach, whereas a combination of both was conducted less often. Regarding the complication rates of our study cohort, our results are in line with the findings of the metaanalysis conducted by Texakalidis and colleagues, hence there was no statistical difference between patients with different fistula subtypes and vascular approaches.

The overall rate of complications in our study cohort was 10.2% (6/59) with an intraprocedural-related complication rate of only 5.1% (3/59). When looking at the tendentially higher complication rate of the combined approach in our study cohort (2/6, 33.3%) it should be considered that these patients presented with complex pathoanatomic conditions that required in almost all cases two therapeutic sessions. The complication rate in our study cohort revealed a comparable level in regard to similar studies with a wide range of ~2–53%; however with a tendency for decrease in the recent years (27, 29).

In only one case access to the cavernous sinus was established through a direct surgical transorbital route because *via* the conventional transarterial and transvenous approach a satisfactory access to the indirect CCF was not possible. Although Chen et al. reported in their review promising results of the transorbital approach for CCF occlusion in specific scenarios, where different pathoanatomic circumstances prevent a conventional route, this approach is accompanied with a more invasive character and potential major local complications such as orbital hemorrhage or globe puncture, so that it currently remains not the first choice when treating dural cavernous fistulas endovascularly (30, 31).

Limitations of our study are its retrospective single-center design and the small number of cases due to its rare etiology. Interobserver variability could distort the post therapeutic angiographical evaluation because not all interventional procedures were executed by the same neuroradiologist.

## Conclusion

This single center study shows endovascular treatment of CCF to be safe and effective in a multidisciplinary experienced center. There was no significant difference of peri-/intraprocedural complications and angiographically and clinical outcome regarding different endovascular treatment options and fistula types. The direct transorbital approach was rarely necessary in our cohort but may be helpful as a bail out strategy. Although complication rates were low, further data and research are required to improve the periprocedural morbidity.

## Data availability statement

The original contributions presented in the study are included in the article/Supplementary material, further inquiries can be directed to the corresponding author.

## Ethics statement

The studies involving human participants were reviewed and approved by Ethics Committee of University Duisburg-Essen (Application number: 21-10240-BO). Written informed consent for participation was not required for this study in accordance with the national legislation and the institutional requirements. Written informed consent was not obtained from the individual(s) for the publication of any potentially identifiable images or data included in this article.

## Author contributions

GA: conceptualization, formal analysis, investigation, writing–original draft, visualization, and methodology. MO: supervision, writing—review and editing, data curation, and conceptualization. YL: data curation and conceptualization. SG: writing—review and editing. MDO: writing—review and editing and formal analysis. BF: supervision and project administration. AE, MF, and MK: investigation. KW: writing—review and editing and investigation. IW: supervision and writing—review and editing. CD: conceptualization, methodology, writing—review and editing, supervision, and project administration. All authors contributed to the article and approved the submitted version.
