# Supplementary material for: Management of carotid cavernous fistulas: A single center experience
Source: Front Neurol. 2023 Feb 9;14:1123139. doi: 10.3389/fneur.2023.1123139 (PMC9947522; doi:10.3389/fneur.2023.1123139)
Supplement: Supplementary file 1 [file Data_Sheet_1.docx]

Supplementary files


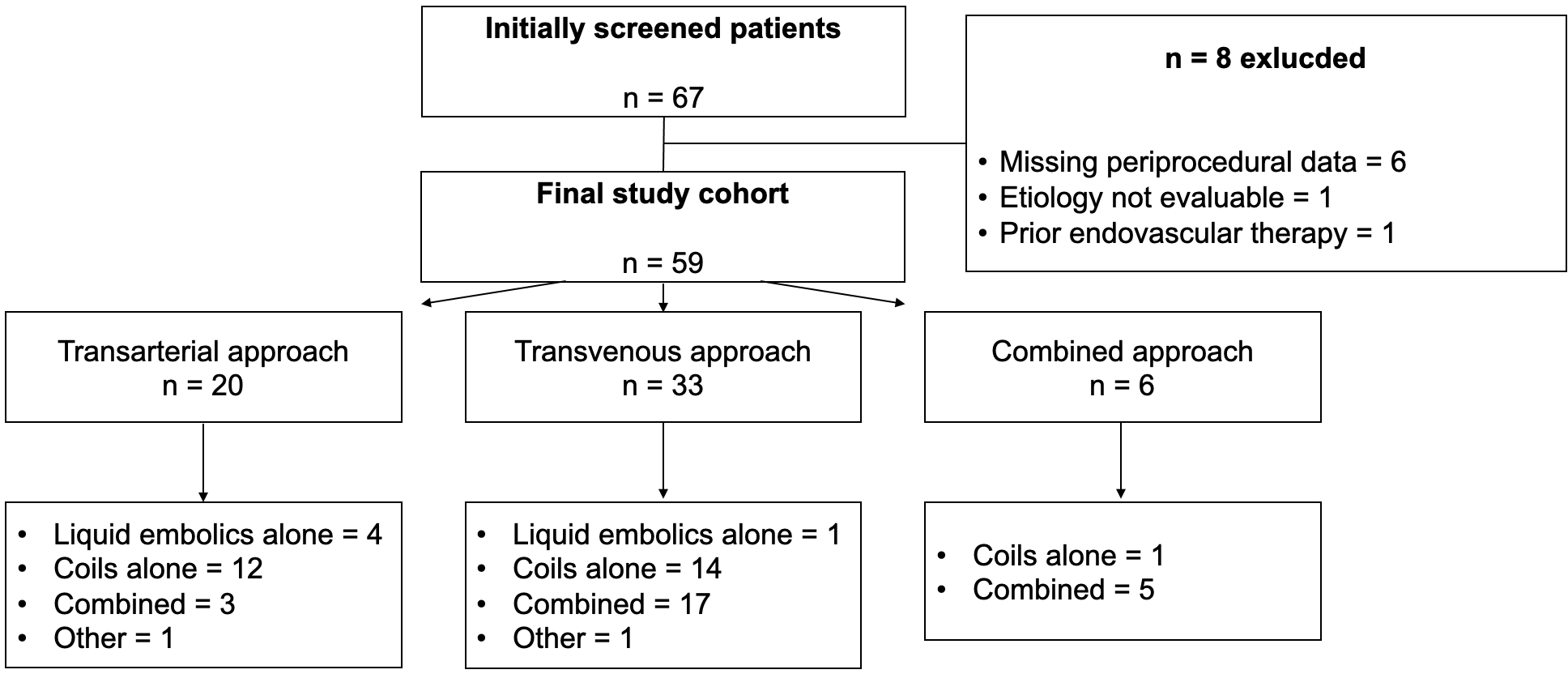


Supplementary file 1: Flow chart of the study cohort. After screening all patients with completed embolization procedure of their Carotid cavernous fistula (CCF) in our institution 59 patients were finally included. Most CCF procedures were attempted via a transvenous approach (n = 33), followed by a transarterial approach (n = 20) or rarely as a combination of both (n = 6). When a transarterial approach was conducted, solely coils were used in the majority of cases (12/20) while the combination of coils and liquid embolics were mostly used in a transvenous setting (17/34).

Supplementary file 2: Description of patients with partial obliteration angiographically

| 1. Combined (transarterial and transvenous) approach to a Barrow Type A CCF with acute traumatic origin. Combination of coils and Onyx – injected under balloon protection – revealed residual fistula flow from the ICA into the SOV. Complicated intraprocedural events with ICA-dissection and intraprocedural M1-occlusion (compare Table 5 for further detail). After TBI patient with tetraplegia and appalic syndrome with further massive protrusion of the ocular bulbi. |
| --- |
| 2. Transarterial approach to a subacute Barrow Type A CCF with minor but residual flow into the fistula via the contralateral ICA after sole coil embolization. Patient showed major relief of symptoms after the intervention. |

CCF = Carotid cavernous fistula, ICA = Internal carotid artery, SOV = Superior ophthalmic vein, TBI = Traumatic brain injury

Supplementary file 3: Detailed description of complications

| ***Periprocedural*** | ***Intraprocedural*** |
| --- | --- |
| 1. Periprocedural pulmonary artery embolism five days postinterventional | 1. Thrombosis of the SOV with consecutive bulbar protrusion and Resolution in the follow up |
| 2. Intraprocedural cardiac arrest with immediate interruption after atropine application  (no postinterventional residuum) | 2. ICA-dissection (cervical segment) and intraprocedural M1-occlusion with complete recanalization after single aspiration  (no infarct demarcation in the postinterventional cross sectional imaging) |
| 3. Postinterventional active femoral bleeding requiring transfusion | 3. Intraprocedural M2-occlusion with only partial recanalization after application of 9mg of Abciximab and 40mg Alteplase |

ICA = Internal carotid artery, SOV = Superior ophthalmic vein

Supplementary file 4: Complication rate in regard of fistula morphology and vascular access

|  | **Entire Cohort**  **n = 59** | **p value** |
| --- | --- | --- |
| ***Fistula morphology*** |  |  |
| Direct (n = 25) | 2 (8.0) | 0.64 |
| Indirect (n = 34) | 4 (11.8) |  |
| ***Approach*** |  |  |
| Transarterial (n = 20) | 1 (5.0) | 0.13 |
| Transvenous (n = 33) | 3 (9.1) |  |
| Combined (n = 6) | 2 (33.3) |  |
